# Col. Martin, on Destroying the Stone in the Bladder

**Published:** 1799-04

**Authors:** 


*As the following communication has been tranſmitted to us through ſuch reſpectable channels, and contains an account of a very extraordinary circumſtance, we are happy in having an opportunity of laying it before our readers, and wait with impatience for the additional information that is expected from Colonel* Martin.

INTRODUCTION.

Sir J. Sinclair having accidentally heard of the very extraordinary method by which Col. Martin, in the ſervice of the Eaſt-India Company, had cured himſelf of a ſtone in the bladder, was thence led to requeſt the Colonel would have the goodneſs to ſend him an account of the proceſs, which he did accordingly, in a letter, of which the following is a copy, in the the words of the writer. Some apology may be made for the language, Col. Martin being a foreigner. A fuller account is soon expected, which will be communicated to the public when received.

*To* Sir *John* Sinclair, *Bart.*

Dear Sir,

I Have been honoured with your very kind favour of the 20th and 22d of December, 1796, as also the one of the 4th of July, 1797. I received them in their proper time, but I have been so much employed, that I had not a proper moment to myself, to answer them as I wished; even now, I cannot do it, being just recovered from a very severe attack of gravel, a circumstance that has not happened to me before, since I have been so fortunate as to cure myself of the stone; which cure was, and must certainly appear very extraordinary, to those who do not know how I accomplished it. I have been looking for a copy of a relation of it, which, I think, I sent to Sir Joseph Banks some time ago, but have not been able to find it, I have only found an old book of memorandums, made during my illness, as I was obliged to make memorandums even of what I was eating and drinking, in order to be able to know what did or did not agree with me. This book is a kind of journal of my disorder, to copy which would be very long; but if Sir Joseph Banks has lost the paper, or the file I sent him, I will make an abstract of the process I used from my memorandums, and also send you one of the files.—I began to file the stone in the bladder, in April, 1782, and as appears by a note I received from Doctor Rennet Murchison, who was at this place as surgeon to the Resident, I soon made an impression upon the stone, and brought away many small pieces, which are in my possession. One of these pieces I sent to Dr. Murchison for his inspection.

His answer was:—

"*Dear Martin, I have examined the Stone with a good microscope; it seems to have a solid shell on the external part, but is internally of a loose texture.—From this appearance, I fancy your mechanical plan has had some effect; but, my dear friend, do not suffer yourself to be so sanguine in your hopes, as to use your file too often, for an inflammation of the bladder might now prove fatal; however, as the internal texture of the stone is loose, and as you have broken the hard surface on the outside, I have no doubt but you may get a great deal of it away, by the cautious use of your instrument.*

"*Your's affectionately,*

June 5, 1782. "R. MURCHISON."

This good man, Dr. Murchison, endeavoured to dissuade me from going on, but as I found daily the good effect of my filing, and never suffered particular pain in doing it; I perſevered till the middle of October of the ſame year, and I think I filed on an average, at the rate of at leaſt three times in the twenty-four hours; I was at firſt puzzled how to bring the ſtone to the neck of the bladder; but I contrived to inject warm water into the bladder, which, endeavouring to diſcharge, it protruded the ſtone to its neck; I then introduced my file between the fleſh and the ſtone, keeping my body inclined againſt a wall all the time, till, by a bad ſtroke, I puſhed the ſtone from the neck of the bladder. In this caſe I could not proceed with filing, but I was obliged to wait till it came again to obſtruct the urine, or it was brought back by injecting warm water into the bladder. Fear of inflammation I had none; for it once happened that a ſpaſm of the whole urethra fixed my file ſo firmly that I could not move it. This ſpaſm laſted about ten minutes, and when relaxed, a great deal of blood came away, and alſo many ſmall pieces of the ſtone. In a couple of days I could renew my filing without any pain, which convinced me that there was no fear of inflammation, and ſuch ſpaſms happened often without any bad conſequence. I had long before made uſe of Mr. Daran's bougies, not knowing that my diſorder was the ſtone, but thought it to ariſe from ſtricture or caruncles at the neck of the bladder. I had alſo made great uſe of lime-water and ſoap, and even injected them into the bladder, when I had found out, that my complaint was the ſtone in the bladder.

Dr. Murchiſon had given me for a long time ſalt of tartar and vitriolic acid; which, I think, did me more hurt than good; as it had once reduced me ſo very low as to be near dying; and, I think, if I had not quitted that plan, againſt the advice of all my friends, and of ſome who are now in England, particularly Mr. Nathaniel Middleton, of Stafford-Place, London, who adviſed me to follow that courſe, I am convinced I ſhould not have lived twenty days. It had reduced me ſo very much, that I was not able to keep myſelf on my legs, or even in an erect poſture without being giddy. In ſpite of advice, I took a large doſe of tartar emetic, and voided a moſt corroſive acid from my ſtomach; from that moment I could ſleep, and keep myſelf on my legs, though reduced to a ſkeleton. After this, I brought my ſtomach into better order, and when well, except the pain of the ſtone, neceſſity, the mother of all invention, ſuggeſted to me the file, which effectually cured me: and I have been extremely well ever ſince, except about two months ago, I was attacked with a fit of the gravel, of which I am now clear; but the neck of the bladder was excoriated, which prevented me from taking my uſual ride, which I otherwiſe do every day, and gallop about eight or ten miles before breakfaſt, and the ſame in the afternoon. Notwithſtanding all theſe exerciſes, I tend to form ſo much acid in my ſtomach, that it would give me the gout if I was to omit them. I will by and and by ſend you a better deſcription of the means by which I cured myſelf, and alſo ſend you one of the files.

You may ſee by my bad Engliſh, that I am deficient, perhaps, in expreſſing myſelf as I wiſhed, ſo that I fear the explanation of my cure may not be ſo clear as if written by one well verſed in Engliſh. I hope to be excuſed for any incorrectneſs in the ſtyle, ſincerely wiſhing that my method might relieve others, as it has done me. To every one of the faculty who wiſhed to know what I did, I have explained it. I am convinced that all perſons may cure themſelves, as it requires very little addreſs. I do not think it poſſible for another to operate, as none but the patient can know where it pains him, and he will naturally know when and how he can introduce the file, as he cannot do it to any purpoſe, but when the ſtone is at or near the neck of the bladder. The file being ſo very ſmall (not thicker than a ſtraw), is eaſily introduced between the fleſhy part and the ſtone, and the motion in filing does not extend beyond the length of about half an inch; and when he does it, he naturally will take care not to puſh the ſtone into the bladder, as he will for this reaſon find it neceſſary to raiſe the file againſt the fleſhy part, and then puſh the file in about half an inch, taking care to avoid touching the ſtone; and when ſo far, to draw and preſs the file upon the ſtone, two motions that are performed very quickly; and as the file is made or cut in ſuch a manner, as to file only in drawing it, and preſſing the file on the ſtone, he cannot avoid filing that part of the ſtone; and, by doing it often in the day and night, muſt at laſt deſtroy it, as I did. The pain I ſuffered from that complaint had made me abandon drinking any liquor but plain water, and I had been ſo much reduced, having at the ſame time ſome ulceration near the neck of the bladder, that I had alſo been obliged to lay aſide the uſe of ſalt and ſpices in my food, to avoid acridity in my urine. My food was nothing elſe but boiled or roaſted meat, and water for my drink, taking care to keep my body open by gentle laxatives. But, as ſoon as the urine became clear, my ſtomach began to be better, and I grew more eaſy, and more regular in filing the ſtone, which I did very often in the day and night, ſometimes ten or twelve times in the day, and paſſed almoſt every day ſmall pieces, till the whole came away; and, as I ſaid above, I have been very well ſince; never had any pain, or return of ſtone or gravel till very lately. But, from the preceding complaint, my ſtomach ſuffered ſo much, that I am very ſubject to form acid; and when the acid prevails, it affects my left kidney, and occaſions me to make water oftener than uſual.

I have lately experienced ſome attacks of gout, which laſt about ſeven or nine days, and a doſe of tartar emetic often cures me, with the aſſiſtance of horſe-exerciſe. All fermented liquors, or liquors containing acidity, as alſo fruit, fruit, or green vegetables, do not agree with me. Animal food and water are my general food and beverage, and I have always been well during the uſe of them.

I have had the pleaſure to receive the printed paper you have been ſo kind to ſend me. Your labours for the improvement of agriculture are very great, and I am extremely happy to learn your ſucceſs. I ſhould be more happy, if I were able to contribute toward ſo uſeful a branch of ſcience; but I fear nothing can be learned in this country; the ſoil is ſo very rich, that every thing vegetates without labour or art. On theſe accounts, the neceſſity of invention does not exiſt.

Excuſe me, my dear ſir, for ſo long a letter: I at firſt propoſed a ſmall one, in anſwer to yours, but I found the ſubject of my late indiſpoſition has carried me ſo far, to make you better acquainted with the means by which I cured myſelf. Believe me, moſt ſincerely,

My dear ſir, your moſt obedient, &c.

*Lucknow, July* 9, 1798. COL. MARTIN.

The above letter being communicated to Mr. HASTINGS, by Sir John Sinclair, he returned an anſwer, of which the following is an extract:—

*To Sir* JOHN SINCLAIR, *Bart.*

Dear Sir, *Daylesford Houſe, Feb.* 28, 1799.

I return you many thanks for the peruſal of Colonel Martin's curious letter; for curious and intereſting it is, even to me, who well remember all the particulars of his caſe, as he has detailed it to you, and even the language in which he has delivered them. The letter from Dr. Murchiſon, a very ſenſible and able practitioner, I well remember. I mentioned the fact once to Mr. Pott, who evidently ſhewed, by his looks and ſilence, that he did not believe it.

I have the honour to be, &c.

WARREN HASTINGS.

